# Endovascular Treatment Combined With Standard Medical Treatment Improves Outcomes of Posterior Circulation Stroke: A Systematic Review and Meta-Analysis

**DOI:** 10.3389/fneur.2022.694418

**Published:** 2022-04-19

**Authors:** Shuju Dong, Yanbo Li, Jian Guo, Yaxi Luo, Jinghuan Fang, Li Tang, Li He

**Affiliations:** Department of Neurology, West China Hospital, Sichuan University, Chengdu, China

**Keywords:** posterior circulation, ischemic stroke, endovascular treatment, standard medical treatment, outcome, meta-analysis

## Abstract

**Aims:**

Whether endovascular treatment (EVT) can further improve the prognosis of patients with posterior circulation ischemic stroke (PCIS) is unclear. This meta-analysis aims to compare the efficacy and safety of PCIS patients treated with EVT plus standard medical treatment (SMT) and SMT alone.

**Methods:**

We systematically searched for relevant randomized controlled trials (RCTs) and prospective cohort trials in MEDLINE, EMBASE, and the Cochrane Library up to February 2022. The primary outcome was favorable functional outcome of the modified Rankin Scale (mRS) with scores of 0–2 or 0–3; secondary outcomes included successful recanalization rate, intracranial hemorrhage (ICH), or symptomatic intracranial hemorrhage (sICH) after treatment and 90-day mortality.

**Results:**

We identified six studies including 1, 385 PCIS patients (957 with EVT plus SMT; 428 with SMT alone). EVT plus SMT substantially improved 90-day functional outcomes compared with SMT alone [mRS score of 0–2: RR=1.95, 95% CI (1.52 – 2.51), *P* < 0.001; mRS score of 0–3: RR = 1.85, 95% CI (1.49 – 2.30), *P* < 0.001, respectively]. Moreover, compared with SMT, combined treatment significantly improved the rate of successful recanalization [RR = 5.03, 95% CI (3.96–6.40), *P* < 0.001] and reduced 90-day mortality [RR = 0.71, 95% CI (0.63–0.79), *P* < 0.001] despite a higher risk of ICH [RR = 6.13, 95% CI (2.50–15.02), *P* < 0.001] and sICH [RR = 10.47, 95% CI [2.79–39.32), *P* = 0.001].

**Conclusion:**

Low-to-moderate evidence from RCTs and non-RCTs showed that increased ICH and sICH risk of EVT plus SMT did not translate to a higher risk of unfavorable outcomes compared with SMT and could even promote independence at 90 days in a real-world cohort.

## Introduction

Posterior circulation ischemic stroke (PCIS) is caused by blood interruption of the vertebrobasilar arterial system and accounts for approximately 20–25% of all ischemic strokes (1). The most common mechanisms responsible for PCIS are embolism (40%), followed by atherosclerosis (32–35%), and other causes of PCIS include dissection, penetrating small-artery diseases, and other identified or unknown etiologies (2). PCIS represents only 1% of all strokes and 5% of large vessel occlusion (LVO) strokes (3, 4). Despite that, PCIS patients with LVO have an extremely poor prognosis, with a 90-day mortality rate of approximately 35–50%, and the majority of deaths (83%) occur in the hospital (5, 6). PCIS patients have higher mortality than anterior circulation stroke (ACS) patients despite successful revascularization (7, 8).

For PCIS patients, successful recanalization is an independent predictor of a good prognosis (9). Although intravenous thrombolysis (IVT) has been shown to be effective and safe, recanalization rates with IVT remain suboptimal in the setting of LVO (10, 11). Evidence in the ACS suggests that endovascular therapy (EVT) can improve recanalization rates or functional outcomes compared with IVT alone (12–14). And application to patients with PCIS patients appears to similarly improve prognosis in these patients (15–17). However, conclusions regarding the benefit of EVT compared with the conservative treatment in improving the clinical outcome of PCIS patients are still unconfirmed.

Several clinical studies (18–24) and subsequent meta-analyses (25) have shown that the benefits of EVT in patients with PCIS are comparable to those in patients with ACS. Similarly, evidence from several recent studies suggested that patients with PCIS treated with EVT may have higher recanalization rates and better outcomes compared to conservative treatment alone (26, 27). In contrast, other studies have shown that PCIS patients receiving EVT have poorer functional outcomes at 90 days, with a mortality rate of 41.9% (28–30).

Although several meta-analyses have attempted to confirm the additional benefit of EVT on basis of SMT in patients with acute BAO (15, 16, 26), the efficacy and safety of EVT in patients with PCIS remain uncertain due to design and methodological flaws (26, 31). Therefore, we aimed to include the latest research evidence to further evaluate the effectiveness and safety of EVT plus standard medical treatment (SMT) over SMT alone in patients with PCIS and to provide more reliable evidence for clinical decision making in PCIS (6, 24, 32, 33).

## Methods

We followed the Preferred Reporting Items for Systematic Reviews and Meta-Analyses guidelines (PRISMA) (34) throughout the design, implementation, analysis, and reporting of this study.

### Literature Search and Information Sources

We identified relevant articles by searching the Medline, EMBASE, and Cochrane Library databases from the inception dates to February 2022. Search terms were based on keywords and subject terms, as shown below: (“basilar occlusion” OR “basilar artery occlusion” OR “vertebrobasilar occlusion” OR “vertebrobasilar artery occlusion” OR “posterior circulation” OR “posterior cerebral circulation”) AND (“intra-arterial” OR “endovascular” OR “thrombectomy” OR “embolectomy” OR “intervention” OR “intravascular” OR “stent” OR “angioplasty”) AND (“standard medical treatment” OR “standard medical therapy” OR “conventional” OR “antiplatelet” OR “antithrombotic” OR “anticoagulation” OR “thrombolysis”). The detailed search strategies for each database were shown in Supplementary Table S1. Manual searching was conducted by searching conference proceedings, clinical trials, and research registers, including the National Institutes of Health's ClinicalTrials.gov, the Clinical Trial Registry, and the metaRegister of Controlled Trials. To identify further published, unpublished, and ongoing studies, we contacted the corresponding researchers to confirm related information, and our search was limited to English language and human studies.

### Inclusion/Exclusion Criteria

Studies were considered to be eligible for inclusion if they: (1) focused on patients who were diagnosed with acute arterial occlusion in posterior circulation (including intracranial or extracranial vertebral artery, basilar artery, and posterior cerebral artery) or had available data of PICS; (2) were randomized controlled trials (RCTs) or prospective cohort trials; (3) confirmed the diagnosis of posterior circulation occlusion by digital subtraction angiography (DSA), computed tomography angiography (CTA), magnetic resonance angiography (MRA) or ultrasound according to the study or corresponding trial protocol; (4) had identifiable intervention treatment groups and compared SMT (including medications of antiplatelet, anticoagulation or IVT with urokinase or Alteplase(rt-PA), or combinations of these medical treatments) with EVT (including intravascular procedures of stenting, angioplasty, thrombectomy, intra-arterial thrombolysis(IAT) or various combinations of these treatments) plus SMT according to local uniform protocol; (5) had a follow-up duration of at least 3 months; (6) identified the outcomes including the modified Rankin Scale score, artery revascularization after surgery, death, and hemorrhage. Studies were excluded if (1) they were case reports, editorials, letters, commentaries, and review articles or (2) they did not clearly report focused results.

### Data Extraction and Quality Assessment

Two neurologists (Shuju Dong and Yanbo Li) screened the potential data and independently conducted data extraction and quality assessment for all the relevant studies. If there was a disagreement, the discrepancy was resolved by Jian Guo. First, an initial screening of titles and abstracts was performed to identify potentially interesting papers. After that, the relevant full text was obtained, and eligibility for inclusion was further evaluated. We designed a standardized data extraction table, which included the author or research group, publication year, study area, study time, study design, sample size, intervention procedures, patients' characteristics, follow-up time, endpoints, study quality, etc. The methodological quality of RCTs and prospective cohort studies in the meta-analysis was assessed through the Cochrane collaborative tool (35) and the Newcastle-Ottawa Quality scale (NOS) ranging from 0 (lowest) to 9 (highest) (36). Individual studies with low risk of bias or high NOS scores from 5 to 9 were used for data synthesis.

### Outcomes

The primary outcome was functional independence assessed with a modified Rankin Scale score (mRS range from 0 [no symptoms] to 6 [death]) of 0–2, mainly at 90 days. Some studies also performed mRS assessments on patients with a different range of 0–3. Therefore, we also analyzed mRS scores 0–3 based on the available data from these individual studies. The secondary outcomes were the rate of recanalization according to the modified Thrombolysis in Cerebral Infarction (mTICI) scale score (good: 2b or 3) within 48 h for vessel recanalization, 90-day mortality, intracranial hemorrhage (ICH), and symptomatic intracranial hemorrhage(sICH) after treatment. The presence of ICH was determined postoperatively by follow-up CT or MRI. sICH was defined as evidence of ICH on imaging, and combined with an increase of 4 or more points in the total NIHSS score; or an increase of 2 or more points in a category; or other adverse events leading to surgery, death, life-threatening, or requiring a prolonged hospital stay.

### Statistical Analysis

We used Stata version 15.0 (StataCorp, College Station, Texas, USA) to perform statistical analysis on pooling data. Forest plots were produced to graphically assess relative risks (RRs) and 95% CI values on primary and secondary endpoints based on individual data. Chi-square tests were used for hypothesis testing (*z*-distribution, and an overall *p*-value < 0.05 was considered statistically significant). As the updated version of “Cochrane Handbook for Systematic Reviews to Interventions” states, a fixed-effect meta-analysis is normally interpreted as being the best estimate of the intervention effect and a random-effects meta-analysis may be used to incorporate heterogeneity among studies (37). So, a fixed-effects model (Mantel–Haenszel method) was first used for RR pooling, and then the random-effect model (inverse variance method) was applied depending on the quantification of heterogeneity, and data instability was predicted simultaneously. Once outcomes were all evaluated, the overall quality of the evidence for each outcome was created using the GRADEpro software (Version 3.6 for Windows) based on the GRADE system (38).

Heterogeneity between studies was assessed using chi-square test-based I^2^ statistics (values of 0 −40% represented low heterogeneity, 30–60% represented moderate heterogeneity, 50–90% represented substantial heterogeneity). In addition, to explain sources of heterogeneity, we conduct subgroup analysis, such as study design (RCTs, non-RCTs), year (cut-off point: 2010 and 2015), clinical trial center (single-center, multicenter), sample size (cut-off point: 0, 100, and 1,000), and race (Asian, Caucasian), which were predefined by stratifying original estimates to detect the influence of these variables on the endpoints. We conducted sensitivity analyses to assess the influence of statistical methods and individual studies on the pooled estimates and to explain between-study heterogeneity. The methods included estimate pooling by a random-effects model, influence analysis, and heterogeneity-reducing algorithm (HETRED) analysis. Publication bias assessments were performed by qualitative analyzes using a visual funnel plot.

## Results

### Study Selection, Characteristics, and Quality Assessment

**Figure 1:**
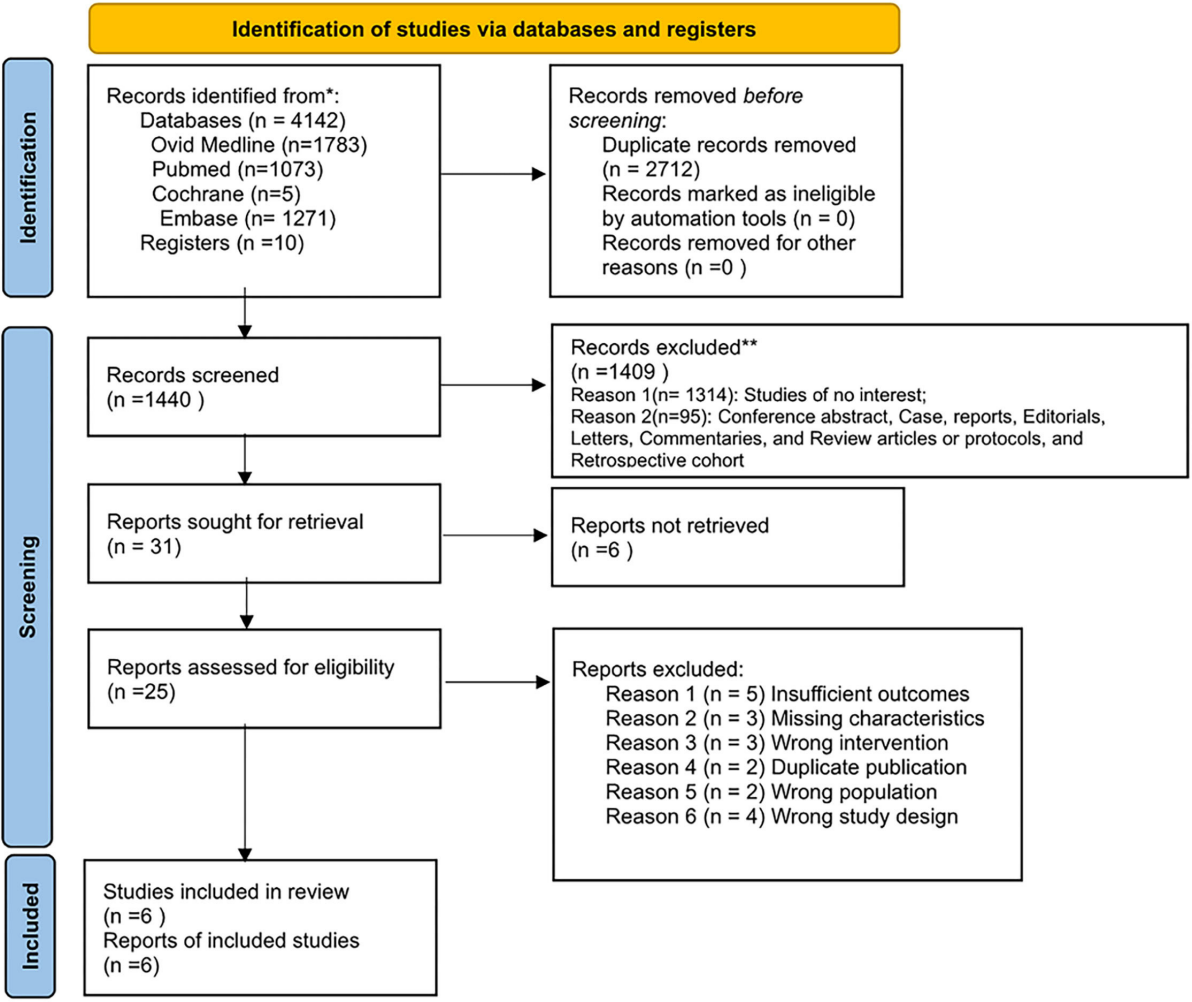
Flow diagram.

Of the 4,152 reports identified in the initial literature search, 2,712 duplicates and 1,955 ineligible studies were removed by screening titles and abstracts, and 25 full-text studies were reviewed. Finally, six of these studies were included in the final analysis (6, 24, 32, 39–41), reporting 957 patients with EVT plus SMT and 428 patients with SMT, including four RCT trials and two non-RCT studies. The flow diagram is shown in Figure 1.

We described the design characteristics and quality assessment results of the six included studies in Table 1. The evaluation results showed that all non-RCTs and RCTs were considered high-quality and had a low risk of bias. The specific details of each scored item are supplied in Supplementary Table S2 and Supplementary Figure S1. More specific information on the six included studies is summarized in Supplementary Table S3. In addition, we provided the clinical characteristics (such as age, sex, National Institutes of Health Stroke Scale (NIHSS) score, cerebrovascular risk factors, and prior stroke or transient ischemic attacks history, etc.) of enrolled patients from RCTs and non-RCTs in Table 2 and Supplementary Table S4, respectively. Table 3 describes some ongoing/unpublished studies in this search.

**Table 1:** Characteristics of included six studies.

**Author, Year**	**Country**	**Study period**	**Design**	**Sample**	**Patients**	**Intervention**	**Control**	**Follow–up (days)**	**Study quality**
**Study**				***n*** **(I/C)**					
Macleod et al. (39) AUST	Australia and New Zealand	Jan.1996 –May 2003	RCT	16 (8/8)	PCIS	IAT + anticoagulation	Anticoagulant	180	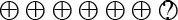
Broussalis et al. (40) Broussalis	Australia	May 2005–June 2012	non–RCT	99 (77/22)	BAO	EVT ± IAT ± IVT	IVT ±Antiplatelet	90	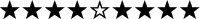
Khoury et al. (41) EASI	Canada	Mar 2013–Oct 2014	RCT	10 (5/5)	BAO + VAO^∫^	EVT + SMT	SMT	90	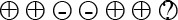
Liu et al. (24) BEST	China	Apr 2015–Sept 2017	RCT	131 (66/65)	BAO + V4	EVT + SMT	SMT	90	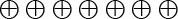
Zi et al. (6) BASILAR	China	Jan 2014–May 2019	non–RCT	829 (647/182)	BAO + V4	EVT + SMT	SMT	90	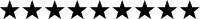
Langezaal et al. (32) BASICS	Brazil etc. 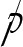	Oct 2011–Dec 2019	RCT	300 (154/146)	BAO + VA (V1, V2, V4)	EVT + SMT	SMT	90	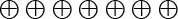

*PCIS, posterior circulation ischemic stroke; BAO, basilar artery occlusion; V4, the V4 segment of vertebrobasilar artery; IAT, intra-arterial thrombolysis; IVT, Intravenous thrombolysis; EVT, endovascular treatment, including intra-arterial medicaments, balloon angioplasty (Aviator, Gateway balloon), implantation of stents (Wingspan, Enterprise) or mechanical clot disruption using a clot retrieval device (Solitaire FR, Penumbra system) would be performed alone or in combination, which followed the American Heart Association/American Stroke Association guidelines or local protocol. SMT, standard medical therapy*.

*The symbol of “⊕ or ⊖ or 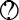
” represented the risk bias of RCT study quality assessment based on Cochrane collaboration's tool, ⊕ represented low risk, ⊖ represented high risk, 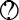 represented unclear risk. 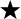 represented the study received one score in nine items of Newcastle-Otawa Quality scale (NOS), 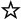 represented the lost score*.

∫ *In EASI study, only the intracranial vertebral or basilar artery was included*.

*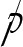 Brazil, Germany, France Italy, the Netherlands, Norway, Switzerland, Czech Republic*.

**Table 2:** Overall clinical characteristics of patients RCT and non-RCT studies.

**Variables**	**RCT patients**		**non-RCT patients**	
	**(Except EASI study)^†^**			
	**Total (*****N =*** **447)**		**Total (*****N=*** **928)**	
	**EVT + SMT**	**SMT**	**EVT + SMT**	**SMT**
	***N =*** **228**	***N =*** **219**	***N =*** **724**	***N =*** **204**
Age, year, Mean ± SD	NA	NA	NA	NA
Male, *N* (%)	109 (47.8)	105 (47.9)	520 (71.8)	143 (70.8)
Atrial Fibrillation, *N* (%)	65 (28.5)	35 (16.0)	NA	NA
Hypertension, *N* (%)	143 (62.7)	131 (49.4)	NA	NA
Diabetes, *N* (%)	44 (20.1)	42 (19.9)	NA	NA
Hyperlipidemia, *N* (%)	6 (8.2)	9 (12.5)	NA	NA
Coronary heart disease, *N* (%)	10 (15.1)	8 (12.3)	NA	NA
Smoking, *N* (%)	24 (32.9)	19 (26.8)	NA	NA
Alcohol, *N* (%)	15 (22.7)	17 (26.1)	NA	NA
Prior stroke or TIA, *N* (%)	28 (12.3)	29 (13.2)	NA	NA
IVT, *N* (%)	139 (63.2)	137 (64.9)	NA	NA
Location of vessel occlusion, *N* (%)				
BA	218 (96.0)	212 (97.2)	601 (83.0)	181 (88.7)
VA	NA	NA	123 (17.0)	23 (11.3)
PCA	NA	NA	0	0
Etiology of stroke				
Atherosclerotic	37 (56.1)	32 (49.2)	418 (64.6)	121 (66.5)
Cardiac embolism	14 (21.2)	17 (26.2)	173 (26.7)	32 (17.6)
Other or unknown	15 (22.7)	16 (24.6)	56 (8.7)	29 (15.9)

*PCA, posterior cerebral artery; BA, basilar artery; VA, vertebrobasilar artery; mRS, modified Rankin score; NIHSS, National Institution of Health stroke scale; TIA, Transient Ischemic Attack; IVT, Intravenous thrombolysis; NA, not applicable*.

† *EASI study was excepted to summary the patients' characteristics due to without available data to extract*.

**Table 3:** Characteristics of related ongoing/unpublished trials.

**Study (ID)**	**Country**	**Estimated study period**	**Design**	**Estimated enrollment**	**Patients**	**Intervention**	**Control**
NCT02157532 EASI	Canada	2013.1–2026.1	RCT	480	Brain large vessel occlusion (including PCS)	SMT + EVT with stent–retriever	SMT
NCT02737189 BAOCHE	China	2016.7–2020.12	RCT	318	BAO	SMT + EVT with stent– retriever	SMT
NCT04177615 RARETBAS	Vietnam	2019.11–2020.12	RCT	109	BAO	EVT + SMT (IVT)	SMT (IVT)
NCT02326428 SITS Open	Sweden	2014.3–2018.1	Non–RCT	341	Acute occlusive stroke (including PCS)	EVT + SMT	SMT

*PCS, posterior circulation stroke; BAO, basilar artery occlusion; IAT, intra-arterial thrombolysis; IVT, Intravenous thrombolysis; EVT, endovascular treatment, including intra-arterial medicaments, balloon angioplasty (Aviator, Gateway balloon), implantation of stents (Wingspan, Enterprise) or mechanical clot disruption using a clot retrieval device (Solitaire FR, Penumbra system) would be performed alone or in combination, which followed the American Heart Association/American Stroke Association guidelines or local protocol, SMT, standard medical therapy, which followed the American Heart Association/American Stroke Association guidelines or local protocol*.

### The mRS Score at 90 Days

**Figure 2:**
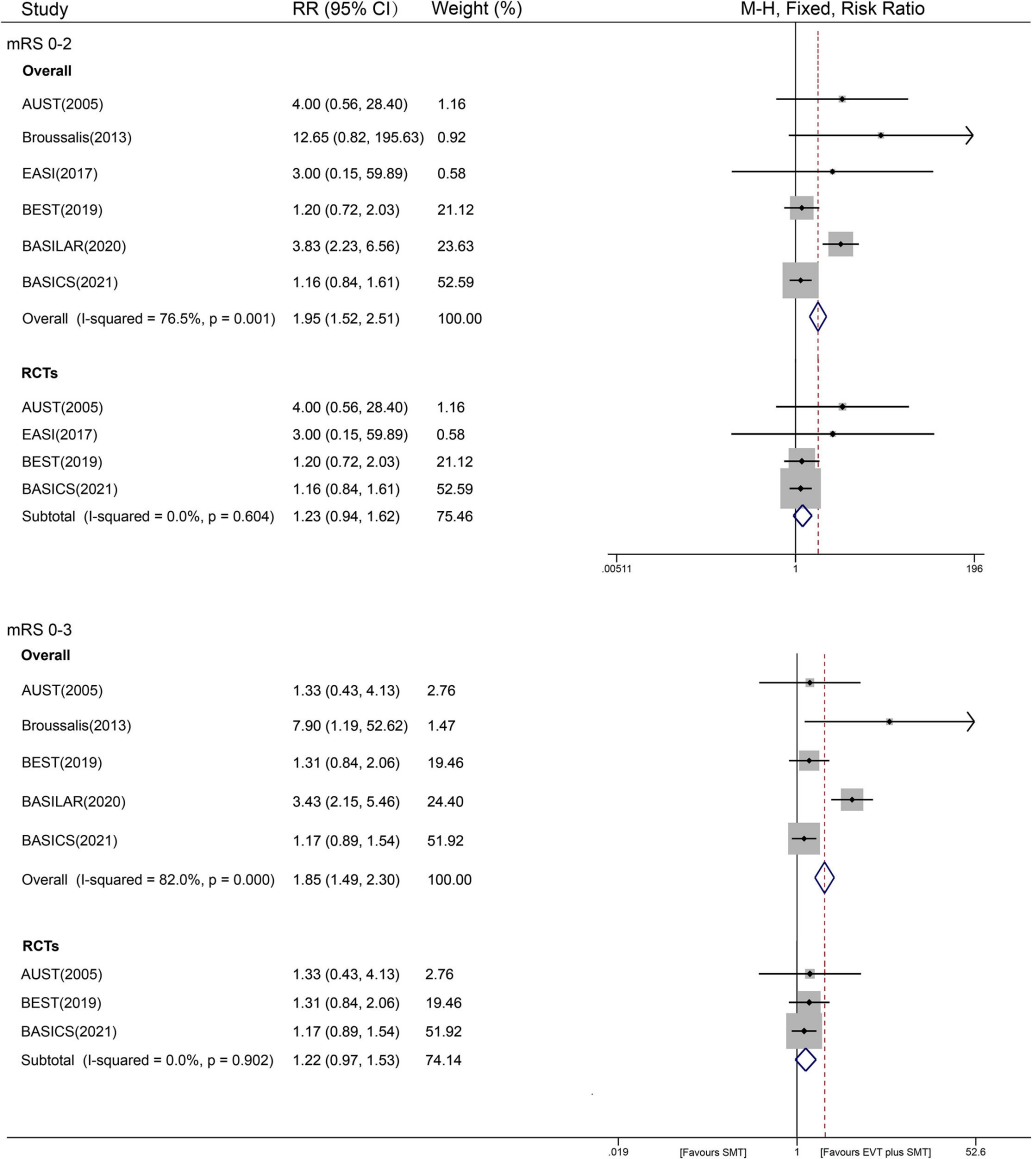
Forest plot of mRS 0–2 or 0–3 with fixed-effect model.

We obtained mRS scores of 0–2 from six studies (1,350 patients), and only five studies reported mRS scores of 0–3 (1,340 patients) at 90 days. Also, we provided the mRS score distribution of included studies, as shown in Supplementary Figure S2. Compared with SMT alone, the combined treatment of EVT and SMT had a better outcome with a mRS score of 0–2 (30.2% vs. 18.1%; *I*^2^ = 76.5%, RR = 1.95, 95% CI [1.52 – 2.51], *P* < 0.001, fixed-effects model). After a stratified analysis according to the study design, the heterogeneity disappeared (Figure 2). The AUST study evaluated the mRS at 6 months after discharge, and all the other studies evaluated the mRS at 90 days. Therefore, we conducted an additional pooled analysis of mRS scores of 0–2 at 90 days, showing a similar result [*I*^2^ = 80.4%, RR = 1.93, 95% CI (1.50 – 2.48), *P* < 0.001]. For different evidence from RCTs and non-RCTs, the RRs with 95% CIs were 1.23 [0.94–1.62] and 4.16 [2.45–7.06], respectively, as shown in Figure 2. The influence analysis of the overall studies indicated that the BASILAR and BASICS studies were aberrant, as shown graphically in Supplementary Figure S3. Then, a random-effects model was added, which showed a 2.07 RR with 95% CI of 1.09–3.92 (*P* = 0.027), similar to the fixed one.

For a mRS score of 0 - 3, the results were similar to an mRS score of 0–2, with an RR value of 1.85 [36.5% vs. 30.0%; 95% CI (1.49–2.30), *P* < 0.001, fixed-effects model], and with significant heterogeneity (*I*^2^ of 82.0%, *P* < 0.001). However, the *I*^2^ was reduced to 0% after subgroup analysis by study design. In the stratified analysis of study design, a favorable trend still existed in non-RCTs, with an RR of 3.68 and 95% CI [2.34–5.79] (*P* < 0.001, fixed-effects model). However, a similar benefit of EVT plus SMT treatment was not found in the RCT analysis, showing an RR of 1.22 [95% CI (0.97–1.53), *P* = 0.096, fixed-effects model] (Figure 2). As stated earlier, a pooled analysis of mRS scores of 0 - 3 on studies excluding AUST was added and showed a similar result as the overall one: RR = 1.87, 95% CI [1.50 – 2.32], *P* < 0.001; *I*^2^ = 86.5%). The result of the influence analysis was consistent with an mRS score of 0–2 (Supplementary Figure S3). A random-effects model was then conducted, and a similar RR of 1.83 with a 95% CI of 1.05–3.19 (*P* = 0.032) was obtained.

### Complete Recanalization Rate

**Figure 3:**
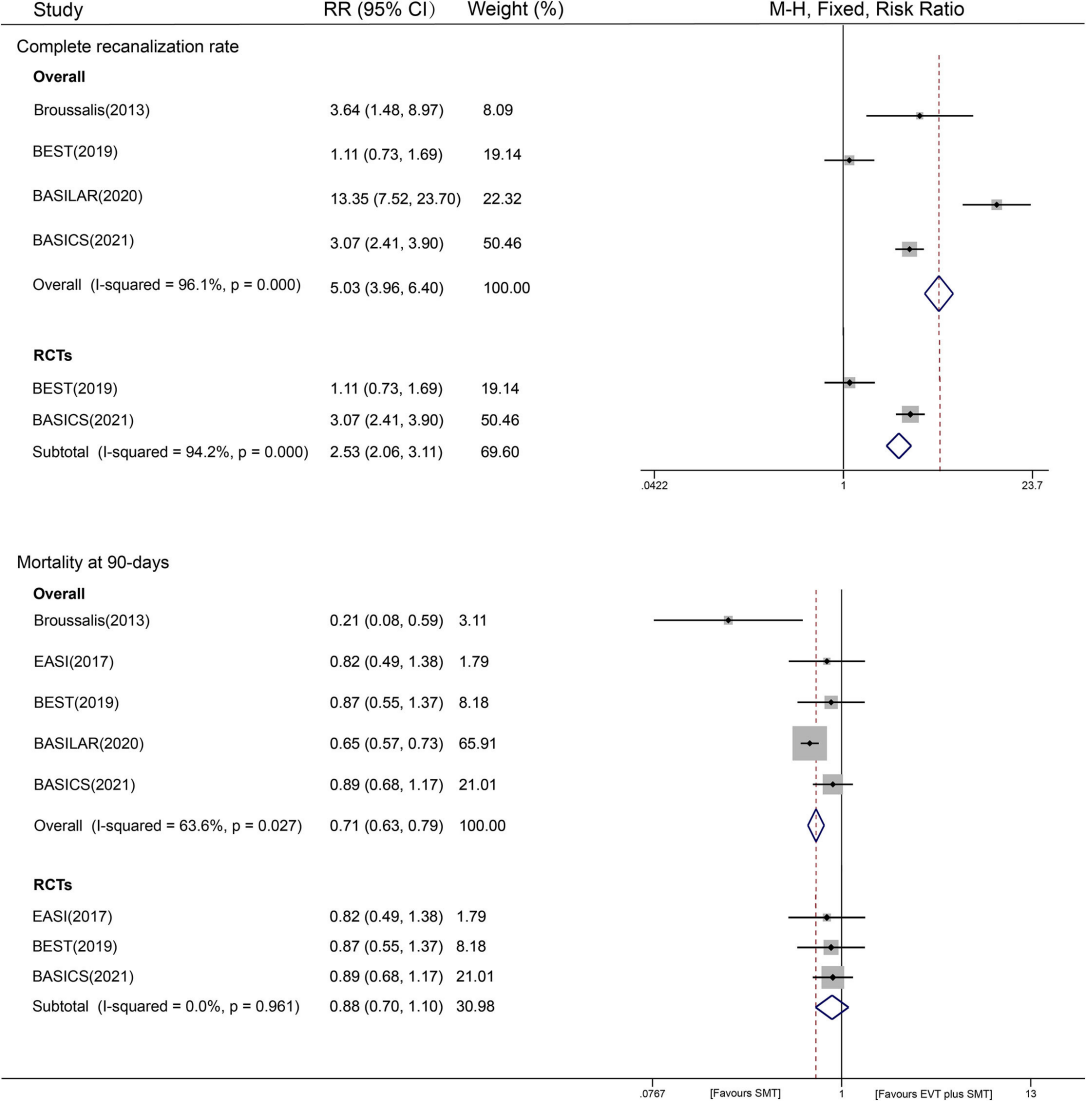
Forest plot of completed recanalization and 90-day mortality with fixed-effect model.

A total of four studies (1,211 patients), including two RCTs and two non-RCTs, reported the recanalization of patients after treatment. We found that compared with using SMT alone, EVT plus SMT significantly increased the complete recanalization rate by approximately 5 times (79.3% vs. 24.8%; RR = 5.03, 95% CI [3.96–6.40], *P* < 0.001, fixed-effects model). When restricted to non-RCTs, the pooled studies showed a similar result, with an RR of 10.77 [95% CI (6.60–17.57), *P* < 0.001, fixed-effects model]. Studies of RCTs had an RR of 2.53 [95% CI (2.06–3.11), *P* < 0.001, fixed-effects model], with *I*^2^ = 94.2% (*P* < 0.001). Substantial interstudy heterogeneity was detected in both non-RCTs (*I*^2^ = 96.1%, *P* < 0.001) and RCTs (*I*^2^ = 94.2%, *P* < 0.001) (Figure 3). In this outcome, all the predefined stratified analyses failed to explain the origin of heterogeneity, showing equally significant heterogeneity (shown in Supplementary Figure S4). The results of the sensitivity analysis predicted an outlier (BASILAR) and a slightly aberrant (BASICS) study (Supplementary Figure S3). Then, a random-effects analysis was introduced, revealing an RR of 3.53 with a 95% CI of 1.41–8.82, and significant heterogeneity was eliminated after the removal of two studies (BASILAR and BEST), with an RR of 3.10 (95% CI 2.46–3.92, *P* < 0.001, fixed-effects model).

### Mortality at 90 Days

A total of five included studies reported 90-day mortality, and we analyzed the mortality risk of 923 patients in the EVT plus SMT group and 411 patients in the SMT group. EVT plus SMT was observed to have a lower mortality risk than SMT alone [41.8% vs. 54.4%, RR = 0.71, 95% CI (0.63–0.79), *P* < 0.001, fixed-effects model]. The heterogeneity among all the studies was apparent, with an I^2^ of 63.6%, *P* = 0.027) (Figure 3).

A subgroup analysis was also conducted to provide evidence from different study designs. The results showed that different therapies were not associated with mortality at 90 days from evidence from RCTs [RR=0.88, 95% CI (0.70–1.10), *P* = 0.544], while EVT plus SMT could reduce the risk of mortality based on evidence from non-RCTs [RR = 0.63, 95% CI (0.55–0.71), *P* < 0.001] (Figure 3). In the sensitivity analysis, we found that the BASILAR trial might be an aberrant study; thus, the RR was repooled in a random-effects model, showing a similar risk of 0.72 [95% CI (0.56–0.94), *P* = 0.015] with an *I*^2^ of 62.9% (*P* = 0.029) after removal. A predefined stratified analysis by year, race, clinical trial center, and the sample size was also conducted, and we found that the factor of publication year may have contributed to the small between-study heterogeneity (restricted to recent five-year publications: *I*^2^ = 50%, *P* < 0.001, Supplementary Figure S5). A HETRED analysis was additionally performed and revealed that removal of two studies (BASILAR and Broussalis) could eliminate heterogeneity (*I*^2^ = 0.0%, *P* = 0.961), with an RR of 0.88 [95% CI (0.70–1.10), *P* = 0.256].

### ICH and sICH After Treatment

**Figure 4:**
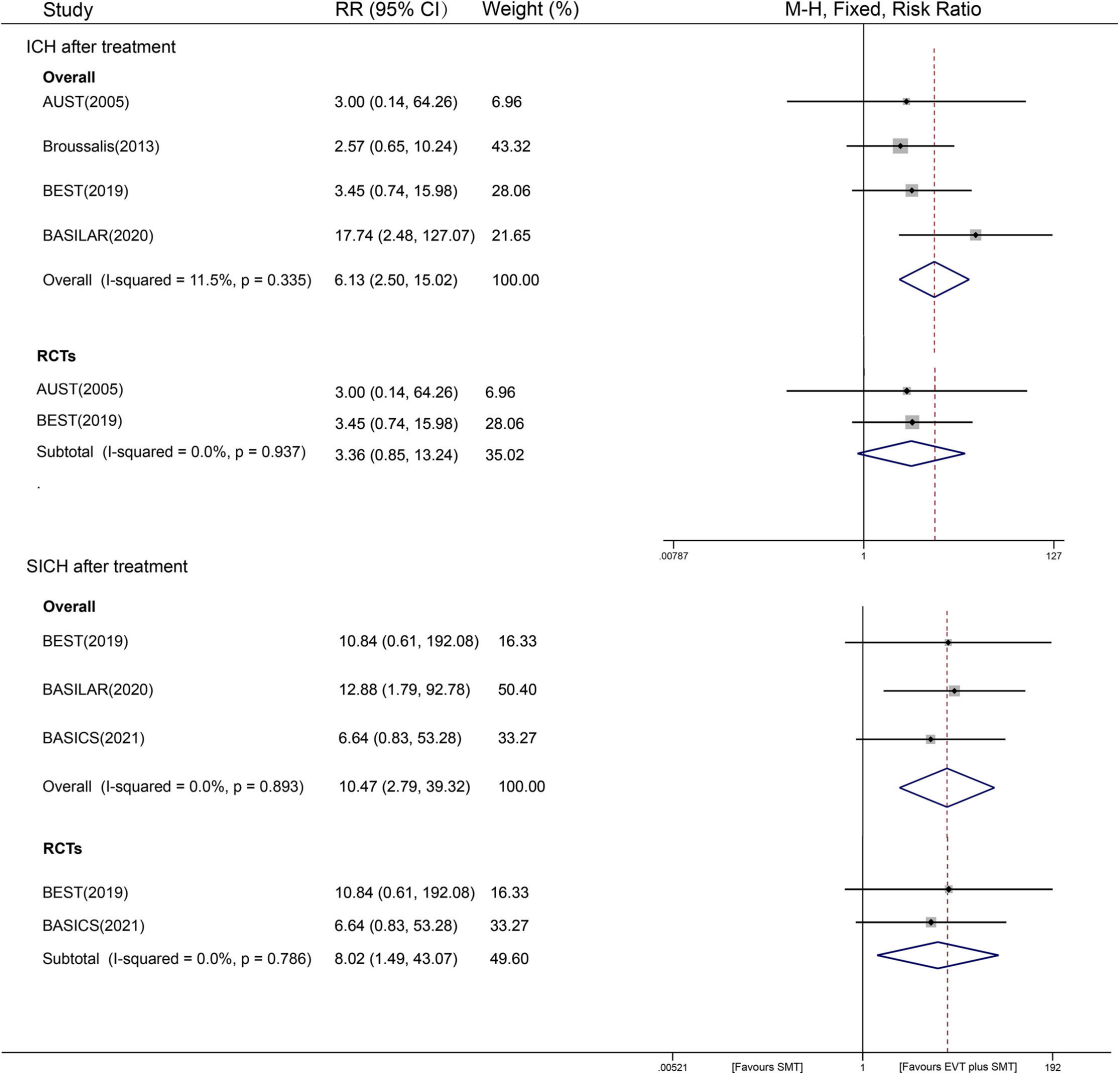
Forest plot of ICH and sICH after treatment with fixed-effect model.

In the secondary outcome analysis of cerebral hemorrhage, four studies collected data on ICH (1, 064 participants), and three studies reported sICH (1, 249 participants) after treatment. In the ICH analysis, we found that combined treatment with EVT showed an increased hemorrhage risk compared with SMT alone [11.2% vs. 1.8%, RR = 6.13, 95% CI (2.50–15.02), *P* < 0.001, fixed-effects model] with low heterogeneity (*I*^2^ = 11.2%, *P* = 0.335). Evidence from different study designs is provided as follows. A subgroup analysis of RCTs showed that the two interventional strategies did not have a significant difference in ICH risk [RR=3.36, 95% CI (0.85–13.24), *P* = 0.083], and no heterogeneity was observed. In two non-RCT studies, a 7.63 times higher risk of ICH was observed in the EVT plus SMT group than in the SMT group [RR = 7.63, 95% CI (2.45–23.72), *P* < 0.001], with a visible between-study heterogeneity of *I*^2^ = 67.6% (*P* = 0.079) (Figure 4). Stratified analyses by other factors also showed that race, year, and different sample sizes may have partially contributed to the between-study heterogeneity (see Supplementary Figure S6). Sensitivity analyses showed that the data of overall studies focusing on ICH events were relatively stable and reliable (shown in Supplementary Figure S3).

For the SICH outcome, the observed risk of EVT plus SMT was 10.47 times that of SMT alone [6.6% vs. 0.5%; 95% CI (2.79-39.23), *P* = 0.001], with an undetected heterogeneity of *I*^2^ = 0% (*P* = 0.738) (Figure 4). The results of sensitivity analyses on SICH outcomes were stable and reliable (shown in Supplementary Figure S3).

### Publication Bias

A publication bias assessment was performed **separately** for each end-point study. The funnel plots showed slight asymmetry, indicating potential publication bias, language bias, exaggerated estimates of flawed method design in smaller studies, or lack of publication of small trials with opposite results. Supplementary Figure S7 shows the funnel plots of mRS scores of 0–2 at 90 days as an example.

### Quality Assessment

The level of evidence for both mRS 0–2 and 0 – 3 outcomes was “low quality” in RCTs and “very low quality” in non-RCTs according to the GRADE approach. As for recanalization rates, the assessment result was “moderate quality” in RCTs and “very low quality” in non-RCTs. Other detailed results of mortality, ICH, and sICH were shown in Table 4.

**Table 4:** The summary of findings table based on GRADE system.

**EVT+SMT compared to SMT for PCIS**						
**Patient or population: PCIS**						
**Intervention: EVT + SMT**						
**Comparison: SMT**						
**Outcomes**	Illustrative comparative risks^*^(95% CI)		**Relative effect (95% CI)**	**No of Participants (studies)**	**Quality of the evidence (GRADE)**	**Comments**
	**Assumed risk**	**Corresponding risk**				
	**SMT**	**EVT + SMT**				
**90-day mRS 0–2 (RCT)** modified Rankin Scale score Follow-up: median 90 days	**281 per 1000**	**346 per 1000** (264 to 456)	**RR 1.23** (0.94 to 1.62)	457 (4 studies)	⊕⊕⊖⊖ **low**^1^, ^2^	–
**90-day mRS 0–2(non-RCT)** modified Rankin Scale score Follow-up: median 90 days	**67 per 1000**	**130 per 1000** (101 to 167)	**RR 1.95** (1.52 to 2.51)	893 (2 studies)	⊕⊖⊖⊖ **very low**^3^, ^4^	–
**90-day mRS 0–3 (RCT)** modified Rankin Scale score Follow-up: median 90 days	**361 per 1000**	**440 per 1000** (350 to 552)	**RR 1.22** (0.97 to 1.53)	447 (3 studies)	⊕⊕⊖⊖ **low**^1^, ^2^	–
**90-day mRS 0–3 (non-RCT)** modified Rankin Scale score Follow-up: median 90 days	**92 per 1000**	**340 per 1000** (216 to 534)	**RR 3.68** (2.34 to 5.79)	893 (2 studies)	⊕⊖⊖⊖ **very low**^2^, ^3^	–
**Complete recanalization rate (RCT)** modified Thrombolysis in Cerebral Infarction (mTICI) scale score ≥ 2b Follow-up: after EVT treatment 24 h	**573 per 1000**	**876 per 1000** (1000 to 1000)	**RR 2.53** (2.06 to 3.11)	288 (2 studies)	⊕⊕⊕⊖ **moderate**^5^	–
**Complete recanalization rate (non-RCT)** modified Thrombolysis in Cerebral Infarction (mTICI) scale score ≥ 2b Follow-up: within 48h h	**74 per 1000**	**792 per 1000** (485 to 1000)	**RR 10.77** (6.6 to 17.57)	288 (2 studies)	⊕⊖⊖⊖ **very low**^4^, ^6^, ^7^	–
**Mortality at 90 days (RCT)** modified Rankin Scale score Follow-up: median 90 days	**431 per 1000**	**379 per 1000** (301 to 474)	**RR 0.88** (0.70 to 1.10)	441 (3 studies)	⊕⊕⊕⊕ **high**	–
**Mortality at 90 days (non-RCT)** modified Rankin Scale score Follow-up: median 90 days	**697 per 1000**	**439 per 1000** (384 to 495)	**RR 0.63** (0.55 to 0.71)	893 (2 studies)	⊕⊖⊖⊖ **very low**^3^	–
**ICH after treatment (RCT)**	**27 per 1000**	**92 per 1000** (23 to 363)	**RR 3.36** (0.85 to 13.24)	139 (2 studies)	⊕⊕⊕⊖ **moderate**^8^	–
**ICH after treatment (non-RCT)** Follow-up: after treatment 24–48 h	**15 per 1000**	**112 per 1000** (36 to 349)	**RR 7.63** (2.45 to 23.72)	917 (2 studies)	⊕⊖⊖⊖ **very low**^4^	–
**sICH after treatment (RCT)**	**5 per 1000**	**38 per 1000** (7 to 204)	**RR 8.02** (1.49 to 43.07)	431 (2 studies)	⊕⊕⊕⊕ **high**	–
**sICH after treatment (non-RCT)** Follow-up: after treatment 48 h	**5 per 1000**	**71 per 1000** (10 to 510)	**RR 12.88** (1.79 to 92.78)	818 (1 study)	⊕⊕⊖⊖ **low**	–

* *The basis for the assumed risk (e.g. the median control group risk across studies) is provided in footnotes. The corresponding risk (and its 95% confidence interval) is based on the assumed risk in the comparison group and the relative effect of the intervention (and its 95% CI)*.

*CI, Confidence interval; RR, Risk ratio*.

*GRADE Working Group grades of evidence*.

*High quality: Further research is very unlikely to change our confidence in the estimate of effect*.

*Moderate quality: Further research is likely to have an important impact on our confidence in the estimate of effect and may change the estimate*.

*Low quality: Further research is very likely to have an important impact on our confidence in the estimate of effect and is likely to change the estimate*.

*Very low quality: We are very uncertain about the estimate*.

1 *Included three RCTs were prematurely terminated because of excessive crossovers or slow enrolment, BASICS expanded the inclusion criteria after 4 years*.

2 *The AUST study evaluated the mRS at 6 months after discharge, and all other studies evaluated the mRS at 90 days*.

3 *Broussalis study has a 21.2% lost to follow-up rate*.

4 *There is a huge difference in the absolute effect between the two studies*.

5 *In all included RCTs, most patients who received SMT were not evaluated for recanalization. Especially in the BEST study, only 14 patients from crossovers were evaluated in the SMT group*.

6 *individual studies adopted different timepoints and approaches to perform the recanalization evaluation*.

7 *Large effect size after consolidation with RR value of 10.77*.

8 *AUST assessed bleeding events on postoperative day 8, whereas the time point for BEST was 24h within randomization*.

## Discussion

The present meta-analysis suggested that there was an overall benefit of EVT in combination with SMT over SMT alone in PCIS. The results supported EVT to improve 90-day functional outcomes, increase successful recanalization and reduce 90-day mortality in PCIS patients, despite the higher risk of ICH and sICH. While the overall 90-day outcome favored EVT, significant heterogeneity was driven by RCTs vs. non-RCTs. The RCT subgroup did not show significant results, but the non-RCTs had a different point estimate of benefit, favoring EVT. This suggested selection and reporting bias in non-RCTs. There was a huge difference in detailed drug medication that thrombolysis rates of RCTs and non-RCTs were, respectively, 80% and 30%. In addition, out of four included RCTs, three were prematurely terminated because of excessive crossovers or slow enrolment, and one (BASICS) (32) expanded the inclusion criteria after 4 years. The beneficial effects of EVT might be partially diluted by the lack of power and performance bias in RCTs. However, although evidence from RCTs indicated a smaller benefit of EVT, there was a much smaller point estimate of RR and 95% CI. Compared with the SMT group, the EVT group of the RCTs generally had more severe strokes on admission, more severe strokes combined with previous strokes or TIA history, and a lower rate of cardiogenic strokes (see in Supplementary Table S4). However, in non-RCTs, the Broussalis study had balanced clinical characteristics between the two groups and the BASILAR study (6) had more advantageous factors associated with good prognoses, such as younger age, higher posterior circulation Acute Stroke Prognosis Early Computed Tomography Score (pc-ASPECTS), etc. (42) in the EVT group. In addition, multimodal treatment existed in the EVT group, including IAT and EVT with aspiration or stent retriever. The recanalization rate varied in different treatment methods, which also led to uncertainty in the EVT effect (9, 15). A meta-analysis suggested that EVT with aspiration can achieve better recanalization and clinical outcomes than EVT with a stent retriever in PCIS patients (43). In addition, 83% of BEST (24) patients and more than half of BASICS (32) patients received EVT with a stent retriever. Furthermore, the small sample of two included RCTs, and stricter inclusion criteria of RCTs might be other potential interpretations for the discrepant results. Also, it was worth considering that the extremely low use of IVT (30%) in both non-RCT studies results in an effectless comparator, which may falsely exaggerate the benefits of EVT. Finally, considering the GRADE evaluation results from RCTs and non-RCTs were low quality and very low quality, respectively, more research evidence was needed to further support EVT can bring benefits.

The degree of recanalization is a critical factor in determining the therapeutic effect and prognosis in AIS (44). EVT plus SMT significantly improved the rate of successful recanalization in both the overall analysis and stratified analysis by study design. However, significant heterogeneity between studies was also detected. In all included RCTs, most patients who received SMT were not evaluated for recanalization. Especially in the BEST study, only 14 patients from crossovers were evaluated in the SMT group. In addition, although the data were insufficient for further subgroup analysis to pinpoint the impact of interventions, evaluation methods, and assessment timepoint of the recanalization on revascularization outcome in a statistically meaningful way, we found that the individual studies adopted different timepoints and approaches to perform the recanalization evaluation. In addition to the factors mentioned earlier, the time from onset to treatment or reperfusion and the proportion of IVT combined use can also be important differences between studies. However, overall, the results of the GRADE evaluation showed that the combined results of RCTs were more certain than those of non-RCTs. Therefore, based on current evidence, combination therapy of EVT with SMT highly correlated with better successful recanalization than SMT alone.

In the overall analysis of mortality at 90 days, we found that EVT probably reduced the 90-day mortality risk compared with SMT alone, with a low–high quality of evidence. This decreasing trend was more pronounced in the non-RCTs and had statistical significance. However, any mortality analysis in an open-label study and non-RCTs should be performed with caution, particularly when the quality of evidence is low. Clinicians may be more aggressive in patients who undergo a procedure. Patients in a non-RCT study may have been conservatively managed because they had a poor prognosis. In addition, although only small heterogeneity was observed, HETERD analysis and stratification were also performed, indicating that BASILAR was a potential source of heterogeneity between studies. By analyzing the clinical features of BASILAR patients, we found that the admission NIHSS score of BASILAR patients was higher than that of other studies, which was closely related to the prognosis of PCIS patients. Overall, the combined evidence indicated that EVT was not associated with an increased risk of death.

Cerebral hemorrhage is one of the most common complications after surgery, and sICH is an independent predictor of poor prognosis in stroke patients with EVT (45). In our meta-analysis, regardless of whether there was symptomatic or asymptomatic ICH, the pooled RR value showed that compared with SMT alone, combined EVT treatment significantly increased the risk of cerebral hemorrhage, with relatively good homogeneity. And for ICH and sICH, the results of the GRADE approach were relatively high-quality compared with other outcomes. The pooled results of sICH from three large samples and recently published studies suggested that the risk of sICH was significantly higher in the real world. In spite of this, the confidence interval of pooled results was obviously wider, and the RR value should be interpreted with caution. In addition, two-thirds of the pooled studies were from Asia, which might represent an overestimated risk of sICH. Overall, the increased risk of a cerebral hemorrhage in the short term did not reverse the benefit of EVT on 90-day favorable outcomes.

In conclusion, thrombectomy has emerged as an excellent candidate for stroke treatment, both in the anterior and posterior circulation. Exploring how to maximize the therapeutic benefit of thrombectomy for patients has been a hot and controversial topic of research. Examples include bridging thrombectomy or direct mechanical thrombectomy (46) and also new combined treatment modalities (arterial thrombolysis after thrombectomy) in the recent research advances (14, 47). Also, with the development of technology and devices for mechanical thrombectomy, distal or isolated posterior cerebral artery occlusion stroke has also been concerned gradually at present (17, 33, 48). As a result, it is possible to think of different options for EVT in combination with SMT, such as the type of drug, the dosage, the combination of different EVT approaches, the sequence of drug therapy vs. the surgical treatment, and the specific type of population to be targeted. In all, more research evidence on the benefits of EVT in PCIS is still needed in the future.

## Limitations

There were also some limitations in our meta-analysis. First, few relevant studies were included in this meta-analysis, and half of the RCTs had a small sample size, 10 patients in the EASI study and 16 patients in the AUST study. In addition, RCTs varied in the local protocol of PCIS patients, as well as neuro-interventional procedures and mechanical devices. In addition, significant heterogeneity was detected between RCTs and non-RCTs, and we failed to stratify the occlusion arteries, stroke etiology, and other potential confounders limited to the original studies' data. These might contribute to a discrepancy in the pooled results for clinical use. Finally, due to the limited number of included studies, we could not conduct meta-regression to further explore the covariates and provide a more in-depth interpretation of our outcomes. And the results of the funnel plots need to be treated with caution. However, this situation will be changed in the future. As far as we know, three related RCTs and one non-RCT are ongoing, and their results will provide more compelling evidence for the efficacy and safety of EVT plus SMT for PCIS.

## Conclusion

In all, the overall low-moderate-quality evidence from RCTs and non-RCTs showed that increased ICH and sICH risk of EVT plus SMT did not translate to higher risks for unfavorable outcomes compared with SMT and could even promote independence at 90 days in a real-world cohort.

## Data Availability Statement

The original contributions presented in the study are included in the article/Supplementary Material, further inquiries can be directed to the corresponding author.

## Author Contributions

SD and YLi contributed to reviewing and screening the related articles for illegible studies and quality assessment, analyzed the data and wrote the draft manuscript. JG contributed to the subordinate design and revision of this manuscript. YLuo contributed to the conception and data analysis for the review. JF contributed to the arbitration of literature evaluation disputes and quality assessment. LT revised the manuscript. LH contributed to conceiving and revising the manuscript. All authors contributed to the article and approved the submitted version.

## Funding

This study was supported by the National Key R&D Program of China (Nos. 2018YFC1311400 and 2018YFC1311401), the National Natural Science Foundation of China (grants. NSFC-81971162), Project funded by China Postdoctoral Science Foundation (Funding number: 2020M673248), and the Fundamental Research Funds for the Central Universities (No. 2020SCU12032, the postdoctoral foundation of Sichuan University).

## Conflict of Interest

The authors declare that the research was conducted in the absence of any commercial or financial relationships that could be construed as a potential conflict of interest.

## Publisher's Note

All claims expressed in this article are solely those of the authors and do not necessarily represent those of their affiliated organizations, or those of the publisher, the editors and the reviewers. Any product that may be evaluated in this article, or claim that may be made by its manufacturer, is not guaranteed or endorsed by the publisher.
