# Supplementary material for: Endovascular Treatment Combined With Standard Medical Treatment Improves Outcomes of Posterior Circulation Stroke: A Systematic Review and Meta-Analysis
Source: Front Neurol. 2022 Apr 19;13:694418. doi: 10.3389/fneur.2022.694418 (PMC9062408; doi:10.3389/fneur.2022.694418)

Supplementary Material

# Supplementary Tables

**There are 5 appendix tables and 7 figures in this appendix file.**

## Supplementary tables

**TableS1 Search strategy**

| **Steps*** | **Queries** |
| --- | --- |
| **Pubmed Search** | ((((((((("Infarction, Posterior Cerebral Artery"[Mesh]) OR basilar occlusion) OR basilar artery occlusion) OR vertebrobasilar occlusion) OR vertebrobasilar artery occlusion) OR posterior circulation) OR Posterior cerebral circulation)) AND (((((((((Thrombolysis) OR anticoagulation) OR antithrombotic) OR antiplatelet) OR Standard medical therapy) OR Standard medical treatment) OR "Thrombolytic Therapy"[Mesh]) OR "Anticoagulants"[Mesh]) OR "Platelet Aggregation Inhibitors"[Mesh])) AND ((((((((((((((angioplasty) OR stent) OR intravascular) OR intervention) OR embolectomy) OR thrombectomy) OR endovascular) OR endovascular) OR intra-arterial) OR "Angioplasty"[Mesh]) OR "Stents"[Mesh]) OR "Embolectomy"[Mesh]) OR "Thrombectomy"[Mesh]) OR "Endovascular Procedures"[Mesh]) |
| **EMBASE Search** | ('basilar artery occlusion'/exp OR 'basilar artery obstruction'/exp OR 'basilar artery insufficiency'/exp OR 'posterior circulation'/exp OR 'basilar occlusion':ti,ab,kw OR 'basilar artery occlusion':ti,ab,kw OR 'vertebrobasilar occlusion':ti,ab,kw OR 'vertebrobasilar artery occlusion':ti,ab,kw OR 'posterior circulation':ti,ab,kw OR 'posterior cerebral circulation':ti,ab,kw) AND ('angioplasty'/exp OR 'stent'/exp OR 'embolectomy'/exp OR 'thrombectomy'/exp OR 'intra arterial':ti,ab,kw OR endovascular:ti,ab,kw OR embolectomy:ti,ab,kw OR thrombectomy:ti,ab,kw OR intervention:ti,ab,kw OR intravascular:ti,ab,kw OR stent:ti,ab,kw OR angioplasty:ti,ab,kw) AND ('anticoagulation'/exp OR 'anticoagulant agent'/exp OR 'standard medical treatment':ti,ab,kw OR 'standard medical therapy':ti,ab,kw OR antiplatelet:ti,ab,kw OR antithrombotic:ti,ab,kw OR anticoagulation:ti,ab,kw OR thrombolysis:ti,ab,kw) AND [english]/lim AND [humans]/lim |
| **OVID(MEDLINE) Search** | 1 basilar occlusion.mp. [mp=title, abstract, original title, name of substance word, subject heading word, floating sub-heading word, keyword heading word, organism supplementary concept word, protocol supplementary concept word, rare disease supplementary concept word, unique identifier, synonyms]  2 basilar artery occlusion.mp. [mp=title, abstract, original title, name of substance word, subject heading word, floating sub-heading word, keyword heading word, organism supplementary concept word, protocol supplementary concept word, rare disease supplementary concept word, unique identifier, synonyms]  3 vertebrobasilar occlusion.mp. [mp=title, abstract, original title, name of substance word, subject heading word, floating sub-heading word, keyword heading word, organism supplementary concept word, protocol supplementary concept word, rare disease supplementary concept word, unique identifier, synonyms]  4 vertebrobasilar artery occlusion.mp. [mp=title, abstract, original title, name of substance word, subject heading word, floating sub-heading word, keyword heading word, organism supplementary concept word, protocol supplementary concept word, rare disease supplementary concept word, unique identifier, synonyms]  5 posterior circulation.mp. [mp=title, abstract, original title, name of substance word, subject heading word, floating sub-heading word, keyword heading word, organism supplementary concept word, protocol supplementary concept word, rare disease supplementary concept word, unique identifier, synonyms]  6 Posterior cerebral circulation.mp. [mp=title, abstract, original title, name of substance word, subject heading word, floating sub-heading word, keyword heading word, organism supplementary concept word, protocol supplementary concept word, rare disease supplementary concept word, unique identifier, synonyms]  7 basilar artery obstruction/  8 vertebrobasilar insufficiency/  9 posterior cerebral artery/  10 1 or 2 or 3 or 4 or 5 or 6 or 7 or 8 or 9  11 intra-arterial.mp. [mp=title, abstract, original title, name of substance word, subject heading word, floating sub-heading word, keyword heading word, organism supplementary concept word, protocol supplementary concept word, rare disease supplementary concept word, unique identifier, synonyms]  12 endovascular.mp. [mp=title, abstract, original title, name of substance word, subject heading word, floating sub-heading word, keyword heading word, organism supplementary concept word, protocol supplementary concept word, rare disease supplementary concept word, unique identifier, synonyms]  13 thrombectomy.mp. [mp=title, abstract, original title, name of substance word, subject heading word, floating sub-heading word, keyword heading word, organism supplementary concept word, protocol supplementary concept word, rare disease supplementary concept word, unique identifier, synonyms]  14 embolectomy.mp. [mp=title, abstract, original title, name of substance word, subject heading word, floating sub-heading word, keyword heading word, organism supplementary concept word, protocol supplementary concept word, rare disease supplementary concept word, unique identifier, synonyms]  15 intervention.mp. [mp=title, abstract, original title, name of substance word, subject heading word, floating sub-heading word, keyword heading word, organism supplementary concept word, protocol supplementary concept word, rare disease supplementary concept word, unique identifier, synonyms]  16 intravascular.mp. [mp=title, abstract, original title, name of substance word, subject heading word, floating sub-heading word, keyword heading word, organism supplementary concept word, protocol supplementary concept word, rare disease supplementary concept word, unique identifier, synonyms]  17 stent.mp. [mp=title, abstract, original title, name of substance word, subject heading word, floating sub-heading word, keyword heading word, organism supplementary concept word, protocol supplementary concept word, rare disease supplementary concept word, unique identifier, synonyms]  18 angioplasty.mp. [mp=title, abstract, original title, name of substance word, subject heading word, floating sub-heading word, keyword heading word, organism supplementary concept word, protocol supplementary concept word, rare disease supplementary concept word, unique identifier, synonyms]  19 angioplasty/  20 stent/  21 embolectomy/  22 thrombectomy/  23 11 or 12 or 13 or 14 or 15 or 16 or 17 or 18 or 19 or 20 or 21 or 22  24 Standard medical treatment.mp. [mp=title, abstract, original title, name of substance word, subject heading word, floating sub-heading word, keyword heading word, organism supplementary concept word, protocol supplementary concept word, rare disease supplementary concept word, unique identifier, synonyms]  25 Standard medical therapy.mp. [mp=title, abstract, original title, name of substance word, subject heading word, floating sub-heading word, keyword heading word, organism supplementary concept word, protocol supplementary concept word, rare disease supplementary concept word, unique identifier, synonyms]  26 antiplatelet.mp. [mp=title, abstract, original title, name of substance word, subject heading word, floating sub-heading word, keyword heading word, organism supplementary concept word, protocol supplementary concept word, rare disease supplementary concept word, unique identifier, synonyms]  27 antithrombotic.mp. [mp=title, abstract, original title, name of substance word, subject heading word, floating sub-heading word, keyword heading word, organism supplementary concept word, protocol supplementary concept word, rare disease supplementary concept word, unique identifier, synonyms]  28 anticoagulation.mp. [mp=title, abstract, original title, name of substance word, subject heading word, floating sub-heading word, keyword heading word, organism supplementary concept word, protocol supplementary concept word, rare disease supplementary concept word, unique identifier, synonyms]  29 thrombolysis.mp. [mp=title, abstract, original title, name of substance word, subject heading word, floating sub-heading word, keyword heading word, organism supplementary concept word, protocol supplementary concept word, rare disease supplementary concept word, unique identifier, synonyms]  30 blood clot lysis/  31 anticoagulation/  32 anticoagulant agent/  33 anticoagulant therapy/  34 24 or 25 or 26 or 27 or 28 or 29 or 30 or 31 or 32 or 33  35 10 and 23 and 34  36 limit 35 to english language  37 limit 36 to humans |
| **Cochrane Search** | #1 (basilar occlusion):kw OR (basilar artery occlusion):kw OR (vertebrobasilar occlusion):kw OR (vertebrobasilar artery occlusion):kw OR (posterior circulation):kw (Word variations have been searched)  #2 MeSH descriptor: [Thrombectomy] this term only  #3 #2  #4 (intra-arterial treatment):kw OR (mechanical thrombectomy):kw OR (endovascular):kw OR (thrombectomy):kw OR (embolectomy):kw (Word variations have been searched)  #5 (intervention treatment):kw OR (intravascular):kw OR (neurointervention):kw (Word variations have been searched)  #6 #3 or #4 or #5  #7 MeSH descriptor: [Drug Therapy] this term only  #8 (Standard medical treatment):kw OR (Standard medical therapy):kw OR (pharma* treatment):kw OR (pharma* therapy):kw OR (Drug Therapy):kw (Word variations have been searched)  #9 (antiplatelet):kw OR (antithrombotic):kw (Word variations have been searched)  #10 #7 or #8 or #9  #11 MeSH descriptor: [Embolectomy] this term only  #12 #2 or #11 or #4 or #5  #13 #1 and #10 and #12 |

*The cut-off time for the search is "current" for February 2022. We have also restricted the type of study to human and English language.

**Table S2 Non-RCTs quality assessment**

|  | Selection | | | |  | Outcome | | | |
| --- | --- | --- | --- | --- | --- | --- | --- | --- | --- |
| First author, year of publication(reference) | Representativeness of exposed cohort | Selection of nonexposed cohort | Ascertainment of exposure | Outcome of interest absent at start of study | Comparability | Assessment of outcome | Follow-up long enough for outcomes to occur | Adequacy of follow-up | Total score |
| Broussalis (2013) | * | * | * | * | * | * | * | * | 8 |
| BASILAR (2020) | * | * | * | * | ** | * | * | * | 9 |

**Table** **S3 Specific characteristic of included six studies**

| Study (Year) | Patients | Diagnosed criteria | Included age | Admission NIHSS | Occlusion location | Onset time | Primary outcome | Second outcome | rate of lost follow-up |
| --- | --- | --- | --- | --- | --- | --- | --- | --- | --- |
| AUST (2005) | PCIS | DSA | 18-85 | without limit | PCA | <24h | Morbidity (Barthel and Rankin Scores) and mortality at 180 days | Recanalization, neurological impairment, safety and tolerability of IAT | 0 |
| Broussalis  (2013) | BAO | CTA/MRA/DSA | without limit | without limit | BA | <12h | mRS and NIHSS at discharge/90days | Recanalization, ICH | 21.2% |
| EASI  (2017) | PCIS | Angiography (as defined in the protocol) | ≥18 | ≥8 | Intracranial vertebral or BA | ≤5h or  clinical-imaging mismatch | mRS 0−2 at 90days,  Mortality at 90 days,  sICH at 24h | Recanalization, adverse events | 0 |
| BASILAR (2020) | BAO | CTA/MRA/DSA | ≥18 | without limit | BA and V4 | <24h | mRS at 90 days | mRS 0−3, Mortality, sICH at 90 days | 0 |
| BEST  (2020) | BAO | CTA/MRA/DSA | ≥18 | without limit | BA and V4 | <8h | mRS 0−3 at 90 days,  Mortality at 90 days | sICH, device-related complications, and other severe adverse events | 0 |
| BASICS  (2021) | BAO | CTA/MRA | 18-85 | ≥10 | BA and VA  (V1, V2, V4) | <6h | mRS 0−3 at 90days,  sICH at 3day  Mortality at 90 days | mRS 0−2 and distribution of mRS at 90days; 24h NIHSS, EQ-5D; PC-ASPECT on CTA or MRA at 24 hours | 0 |

Abbreviations: PCA=posterior cerebral artery, BA=basilar artery, VB= vertebrobasilar artery, V4= the V4 segment of vertebrobasilar artery, IAT= intra-arterial thrombolysis, mRS = modified Rankin score, NIHSS=National Institution of Health stroke scale, ICH=intracerebral hemorrhage, sICH=symptom intracerebral hemorrhage, PC-ASPECT=posterior circulation- Alberta Stroke Program Early CT Score, CTA= computed tomography angiography, MRA=magnetic resonance angiography

**Table S4** **Baseline Clinical characteristics of patients from included studies**

| Variables | Macleod et al., 2005  AUST | | Liu et al., 2019  BEST | | Langezaal et al., 2021  BASICS | | Broussalis et al., 2013  Broussalis | | Zi et al., 2020  BASILAR | |
| --- | --- | --- | --- | --- | --- | --- | --- | --- | --- | --- |
|  | EVT + SMT N=8 | SMT N=8 | EVT + SMT N=66 | SMT N=154 | EVT + SMT N=154 | SMT N=146 | EVT + SMT N=77 | SMT N=22 | EVT + SMT N=647 | SMT N=182 |
| Age, year, Mean±SD | 64.3±11.1 | 63.8±12.3 | 62±18.20 | 66.2±12.9 | 66.8±13.1 | 67.2±11.9 | 68(32-89)^‡^ | 72 (31-94))^‡^ | 64(73) | 67(59-76) |
| Male, N (%) | 7(82.5) | 3(37.5) | 48(72.7) | 54(35.1) | 50(34.2) | 104(34.7) | 37(48.0) | 14(63.6) | 483(74.6) | 129(70.9) |
| Atrial Fibrillation,N (%) | 3(37.5) | 3(42.8) | 18(27.3) | 44(28.6) | 22(15.1) | 66 (22.0) | NA | NA | 136(21.0) | 24(13.2) |
| Hypertension, N (%) | 5(62.5) | 7(87.5) | 45(68.2) | 93(60.4) | 82(56.6) | 175(58.3) | NA | NA | 451(69.7) | 134(73.6) |
| Diabetes, N (%) | NA | NA | 106(15.1) | 34/153(22.2) | 31(21.2) | 65(21.7) | NA | NA | 149(23.0) | 40(22.0) |
| Hyperlipidemia, N (%) | 3/7(42.8) | 4/7(57.1) | 3(4.5) | NA | NA | NA | NA | NA | 214(33.1) | 69(37.9) |
| Coronary heart disease, N(%) | NA | NA | 10(15.1) | NA | NA | NA | NA | NA | 105(16.4) | 27(14.8) |
| Smoking, N (%) | 2/7(28.5) | 0/6(0) | 22(33.3) | NA | NA | NA | NA | NA | 235(36.3) | 42(23.1) |
| Alcohol, N (%) | NA | NA | 15(22.7) | NA | NA | NA | NA | NA | NA | NA |
| Admission NIHSS, Median (IQR)^‡^ | 23 (7–29) | 18 (5–29) | 3(18-38) | 21 | 22 | NA | 22(4-28) | 23(12-28) | 26.5(16-33) | 27(17-33) |
| Prior stroke or TIA,  N (%) | 3(37.5) | 2(25.0) | 14(21.2) | 11(7.1) | 7(4.8) | 18(6.0) | NA | NA | 140(21.6) | 48(26.4) |
| IVT, N (%) | NA | NA | 18(27.3) | 121(78.6) | 116(79.5) | 237(79.0) | 30(39.0) | 0(0) | 119(18.4) | 47(25.8) |
| Onset to treatment, min, Median (IQR) | NA | NA | 59  (29-83) | 120  (84-198) | 138  (96-210) | NA | 259  (60-1080)^‡^ | 314  (90-720)^‡^ | 246  (132-390) | 221  (116-407) |
| Onset to puncture time, min, Median (IQR) | 710 | 749 | 114  (66-150) | 264  (198-372) | NA | 264  (198-372) | NA | NA | 328  (220-493) | NA |
| Onset to reperfusion time, min, Median (IQR) | NA | NA | 400  (269-526) | NA | NA | NA | NA | NA | 441(328-627) | NA |
| Location of vessel occlusion, N (%) | | | | | | | | | | |
| BA | 5/7(71.4) | 6/7(85.7) | 59(89.4) | 60(92.3) | 154(100) | 146(100) | 77(100) | 22(0) | 524(81.0) | 159(87.4) |
| VA | 2/7(28.6)^ƒ^ | 1(14.3) ^ƒ^ | 7(10.6) | 0 | 0 | 0 | 0 | 0 | 123(19.0) | 23(12.6) |
| PCA |  |  | 0 | 0 | 0 | 0 | 0 | 0 | 0 | 0 |
| Etiology of stroke |  |  |  |  |  |  |  |  |  |  |
| Atherosclerotic | NA | NA | 37(56.1) | NA | NA | NA | NA | NA | 418(64.6) | 121(66.5) |
| Cardiac embolism | NA | NA | 14(21.2) | NA | NA | NA | NA | NA | 173(26.7) | 32(17.6) |
| Other or unknown | NA | NA | 15(22.7) | NA | NA | NA | NA | NA | 56(8.7) | 29(15.9) |

Abbreviations: PCA=posterior cerebral artery, BA=basilar artery, VA= vertebrobasilar artery, mRS= modified Rankin score, NIHSS=National Institution of Health stroke scale, TIA=Transient Ischemic Attack; IVT=Intravenous thrombolysis, mTICI= Modified Treatment in Cerebral Infarction, ICH=intracerebral hemorrhage, SICH= symptom intracerebral hemorrhage, NA= not applicable.

† EASI study was excepted to summary the patients’ characteristics due to without available data to extract.

‡ NIHSS score was described as median(range) in AUST study, and the range of NIHSS score was absent in BASICS study.

ƒ These data represented the combined proportion of VA and PCA.

**Table S5 PRISMA 2020Checklist**

| **Section and Topic** | **Item #** | **Checklist item** | **Location where item is reported** |
| --- | --- | --- | --- |
| **TITLE** | | |  |
| Title | 1 | Identify the report as a systematic review. | Line 1-2 |
| **ABSTRACT** | | |  |
| Abstract | 2 | See the PRISMA 2020 for Abstracts checklist. | Line 15-33 |
| **INTRODUCTION** | | |  |
| Rationale | 3 | Describe the rationale for the review in the context of existing knowledge. | Line 35-56 |
| Objectives | 4 | Provide an explicit statement of the objective(s) or question(s) the review addresses. | Line 57-62 |
| **METHODS** | | |  |
| Eligibility criteria | 5 | Specify the inclusion and exclusion criteria for the review and how studies were grouped for the syntheses. | Line 81-94 |
| Information sources | 6 | Specify all databases, registers, websites, organisations, reference lists and other sources searched or consulted to identify studies. Specify the date when each source was last searched or consulted. | Line 67-68 |
| Search strategy | 7 | Present the full search strategies for all databases, registers and websites, including any filters and limits used. | Line 74  Appendix TableS1 |
| Selection process | 8 | Specify the methods used to decide whether a study met the inclusion criteria of the review, including how many reviewers screened each record and each report retrieved, whether they worked independently, and if applicable, details of automation tools used in the process. | Line 96-103  Fig 1 |
| Data collection process | 9 | Specify the methods used to collect data from reports, including how many reviewers collected data from each report, whether they worked independently, any processes for obtaining or confirming data from study investigators, and if applicable, details of automation tools used in the process. | Line 120-142 |
| Data items | 10a | List and define all outcomes for which data were sought. Specify whether all results that were compatible with each outcome domain in each study were sought (e.g. for all measures, time points, analyses), and if not, the methods used to decide which results to collect. | Line 108-118 |
|  | 10b | List and define all other variables for which data were sought (e.g. participant and intervention characteristics, funding sources). Describe any assumptions made about any missing or unclear information. | Line 87-93 |
| Study risk of bias assessment | 11 | Specify the methods used to assess risk of bias in the included studies, including details of the tool(s) used, how many reviewers assessed each study and whether they worked independently, and if applicable, details of automation tools used in the process. | Line 132-142 |
| Effect measures | 12 | Specify for each outcome the effect measure(s) (e.g. risk ratio, mean difference) used in the synthesis or presentation of results. | Line 120-122 |
| Synthesis methods | 13a | Describe the processes used to decide which studies were eligible for each synthesis (e.g. tabulating the study intervention characteristics and comparing against the planned groups for each synthesis (item #5)). | Line 81-94  Line 100-106 |
|  | 13b | Describe any methods required to prepare the data for presentation or synthesis, such as handling of missing summary statistics, or data conversions. | Line 100-106 |
|  | 13c | Describe any methods used to tabulate or visually display results of individual studies and syntheses. | Line 120, 130 |
|  | 13d | Describe any methods used to synthesize results and provide a rationale for the choice(s). If meta-analysis was performed, describe the model(s), method(s) to identify the presence and extent of statistical heterogeneity, and software package(s) used. | Line 120-130 |
|  | 13e | Describe any methods used to explore possible causes of heterogeneity among study results (e.g. subgroup analysis, meta-regression). | Line 132-141 |
|  | 13f | Describe any sensitivity analyses conducted to assess robustness of the synthesized results. | Line 132-141 |
| Reporting bias assessment | 14 | Describe any methods used to assess risk of bias due to missing results in a synthesis (arising from reporting biases). | Line 141-142 |
| Certainty assessment | 15 | Describe any methods used to assess certainty (or confidence) in the body of evidence for an outcome. | Line 129-131 |
| **RESULTS** | | |  |
| Study selection | 16a | Describe the results of the search and selection process, from the number of records identified in the search to the number of studies included in the review, ideally using a flow diagram. | Line 145-151 |
|  | 16b | Cite studies that might appear to meet the inclusion criteria, but which were excluded, and explain why they were excluded. | NA |
| Study characteristics | 17 | Cite each included study and present its characteristics. | Line 147, 152-153 Table2, Appendix TableS3-S4 |
| Risk of bias in studies | 18 | Present assessments of risk of bias for each included study. | Line 153-154  Fig 1, Appendix TableS2 |
| Results of individual studies | 19 | For all outcomes, present, for each study: (a) summary statistics for each group (where appropriate) and (b) an effect estimate and its precision (e.g. confidence/credible interval), ideally using structured tables or plots. | Line 163-236  Fig2-4 |
| Results of syntheses | 20a | For each synthesis, briefly summarise the characteristics and risk of bias among contributing studies. | Line 163-236, Table4 Appendix Fig3-6 |
|  | 20b | Present results of all statistical syntheses conducted. If meta-analysis was done, present for each the summary estimate and its precision (e.g. confidence/credible interval) and measures of statistical heterogeneity. If comparing groups, describe the direction of the effect. | Line 160-238  Fig 2,3,4 |
|  | 20c | Present results of all investigations of possible causes of heterogeneity among study results. | Line 160-238  Appendix Fig3-6 |
|  | 20d | Present results of all sensitivity analyses conducted to assess the robustness of the synthesized results. | Line 160-238  Appendix Fig3-6 |
| Reporting biases | 21 | Present assessments of risk of bias due to missing results (arising from reporting biases) for each synthesis assessed. | Line 238-242  Appendix Fig7 |
| Certainty of evidence | 22 | Present assessments of certainty (or confidence) in the body of evidence for each outcome assessed. | Line 244-248  Table4 |
| **DISCUSSION** | | |  |
| Discussion | 23a | Provide a general interpretation of the results in the context of other evidence. | Line 251-315 |
|  | 23b | Discuss any limitations of the evidence included in the review. | Line 329-340 |
|  | 23c | Discuss any limitations of the review processes used. | Line 329-340 |
|  | 23d | Discuss implications of the results for practice, policy, and future research. | Line 316-326  337-340 |
| **OTHER INFORMATION** | | |  |
| Registration and protocol | 24a | Provide registration information for the review, including register name and registration number, or state that the review was not registered. | NA |
|  | 24b | Indicate where the review protocol can be accessed, or state that a protocol was not prepared. | NA |
|  | 24c | Describe and explain any amendments to information provided at registration or in the protocol. | NA |
| Support | 25 | Describe sources of financial or non-financial support for the review, and the role of the funders or sponsors in the review. | Line 356-359 |
| Competing interests | 26 | Declare any competing interests of review authors. | Line 346-347 |
| Availability of data, code and other materials | 27 | Report which of the following are publicly available and where they can be found: template data collection forms; data extracted from included studies; data used for all analyses; analytic code; any other materials used in the review. | Line 364-365 |

## Supplementary figures

**Appendix Fig S1 Risk of bias summary: review authors' judgements about each risk of bias item for each included study.**


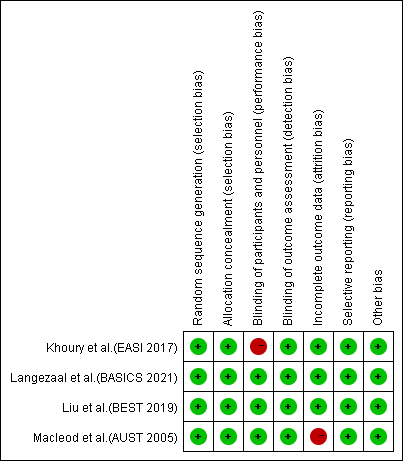


**Appendix Fig S2** **The mRS score distribution of all included studies**


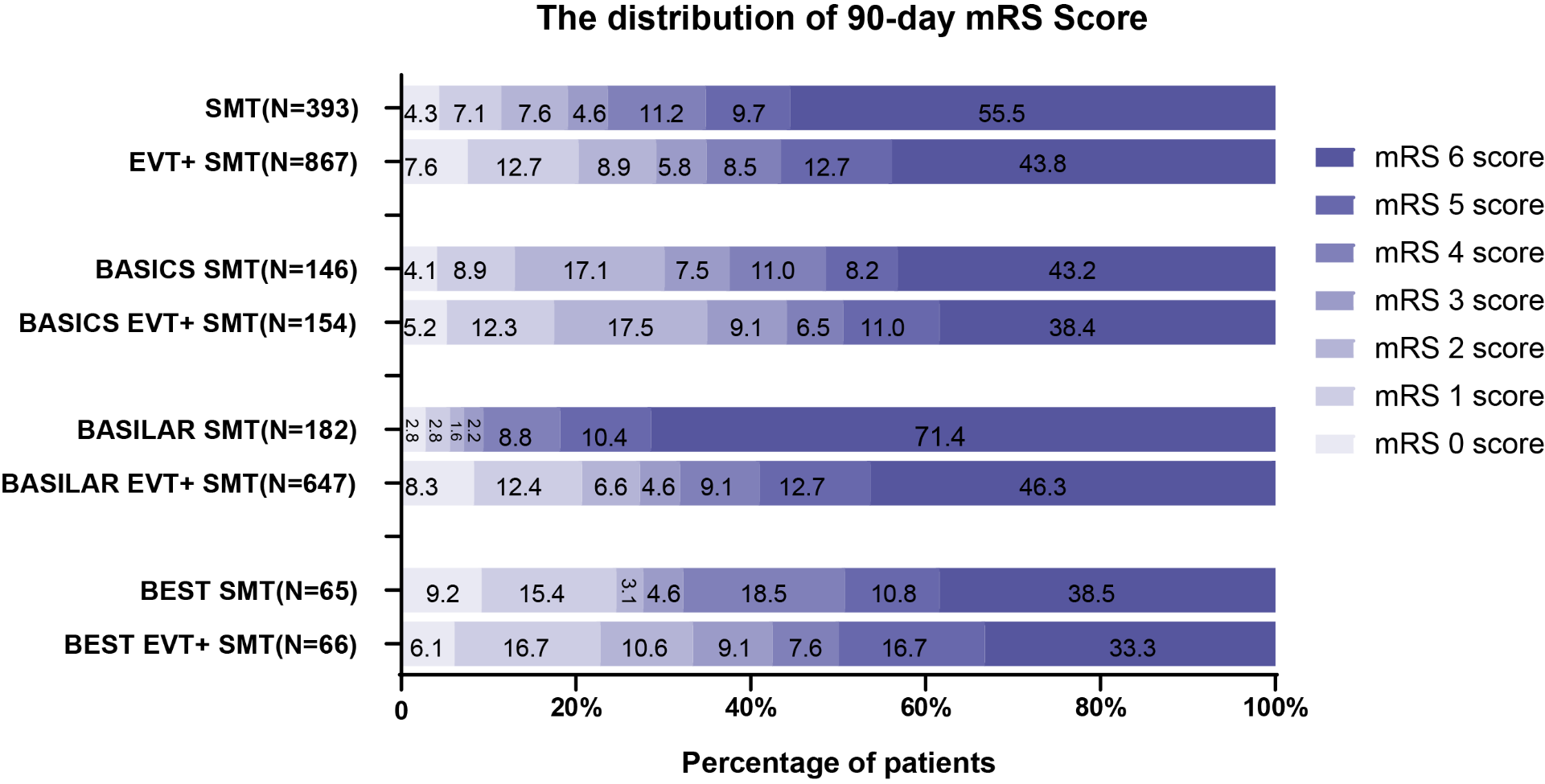


Note: Three of the six included articles had raw data on the distribution of mRS, but the distribution of mRS was not the primary outcome of the article. Therefore, we did not perform a synthetic analysis of this result, only showing the trend of the distribution of the combined mRS.

**Appendix Fig S3 The sensitivity analysis of the included studies for each outcome**

**
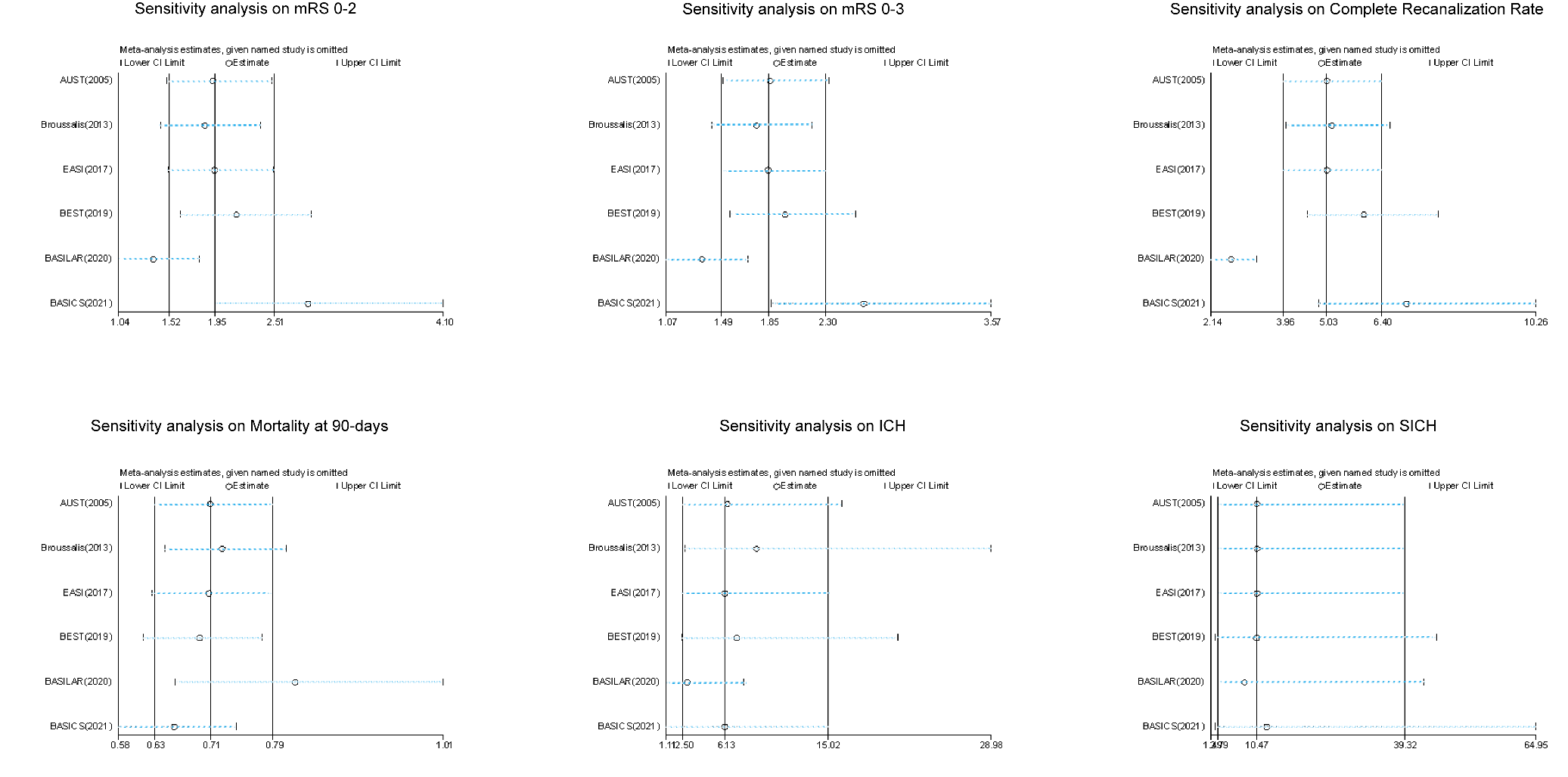
**

**Appendix Fig S4 Stratified analysis by race, published period and sample size on recanalization outcome.**


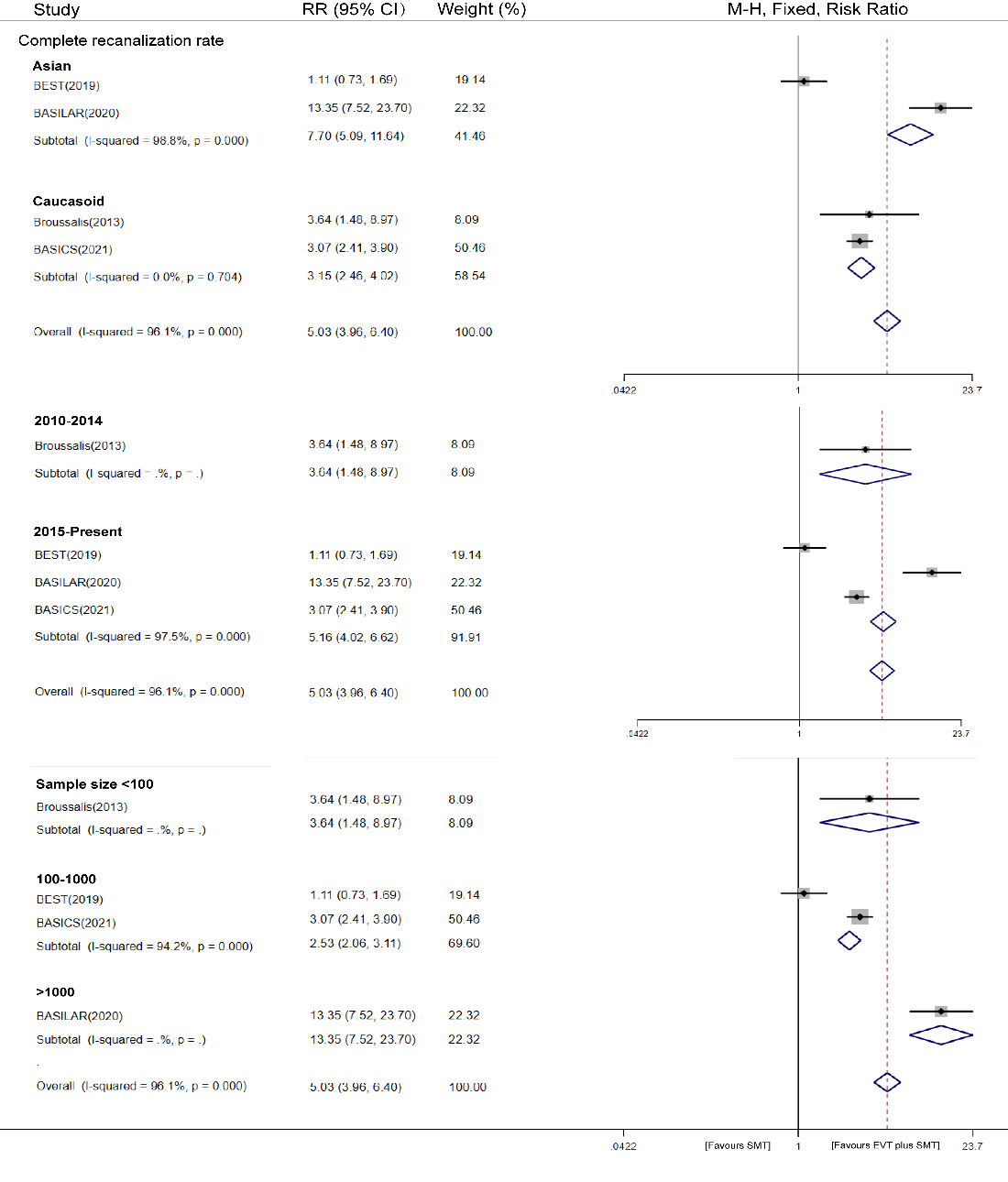


**Appendix Fig S5 Predefined stratified analysis by year, race, clinical trial center and sample size on 90-day mortality**


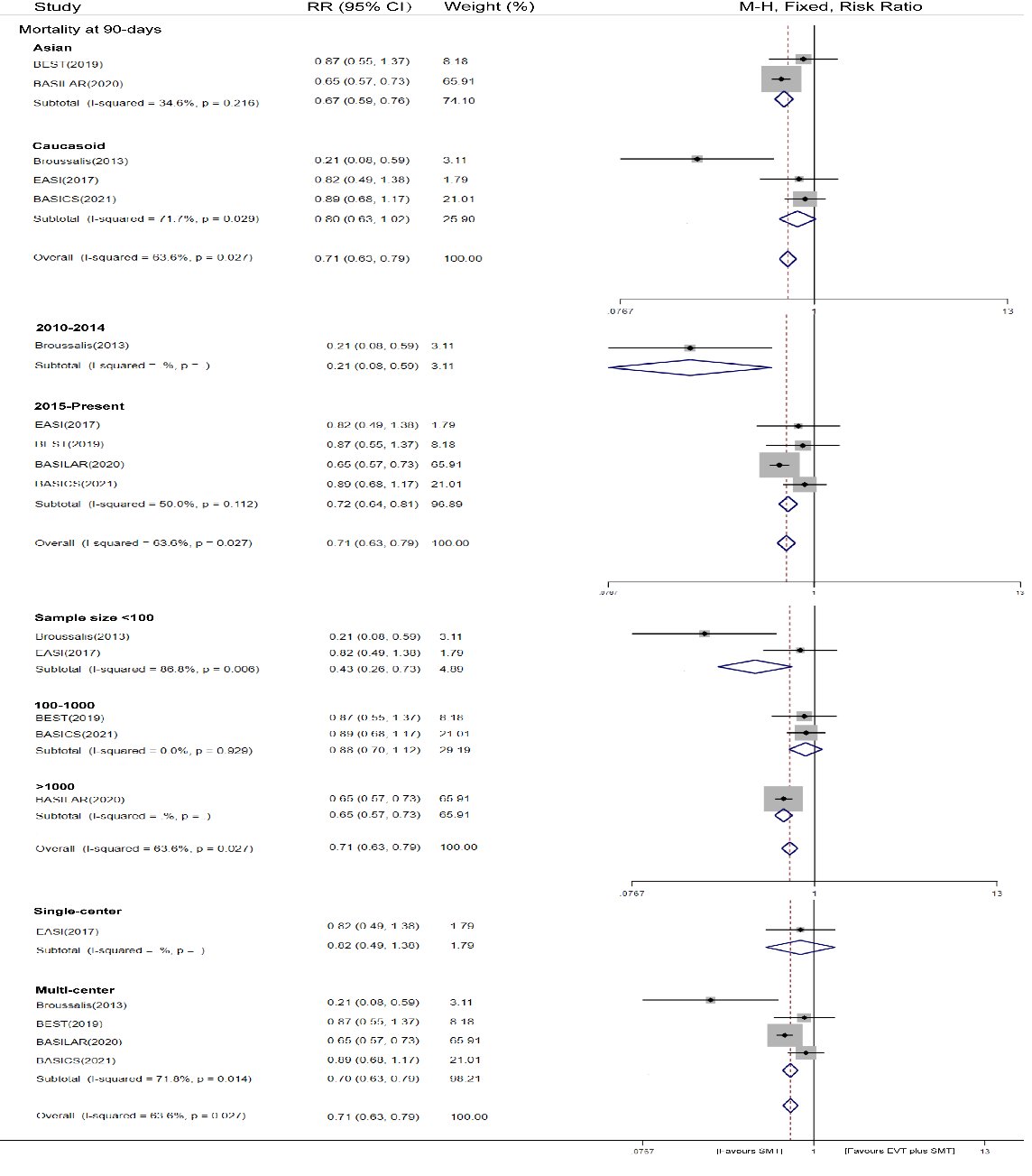


**Appendix Fig S6 Predefined stratified analysis by year, race, clinical trial center and sample size on intracranial hemorrhage after treatment.**


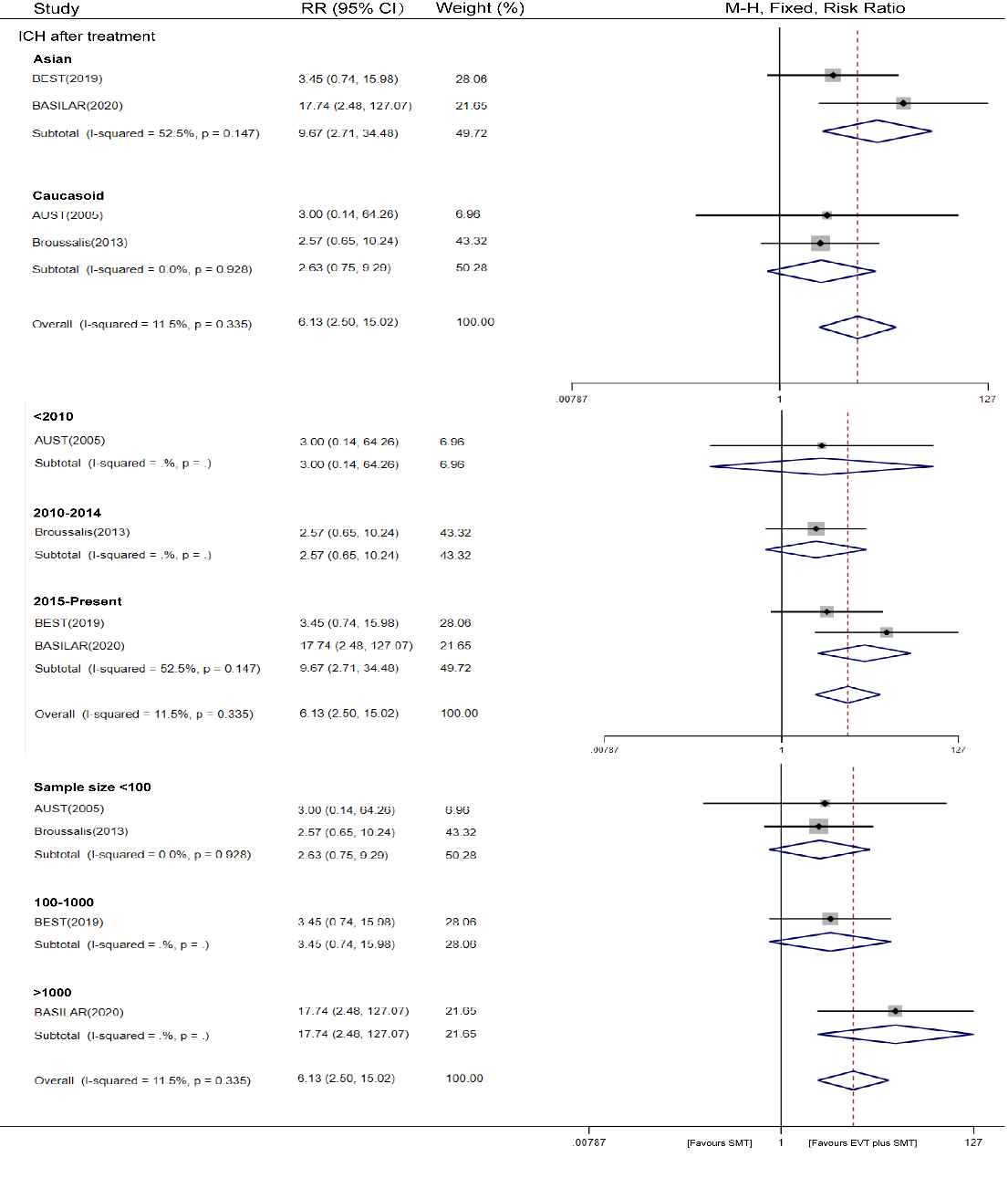


**Appendix Fig S7 The funnel plots of mRS 0-2 at 90 days as example.**


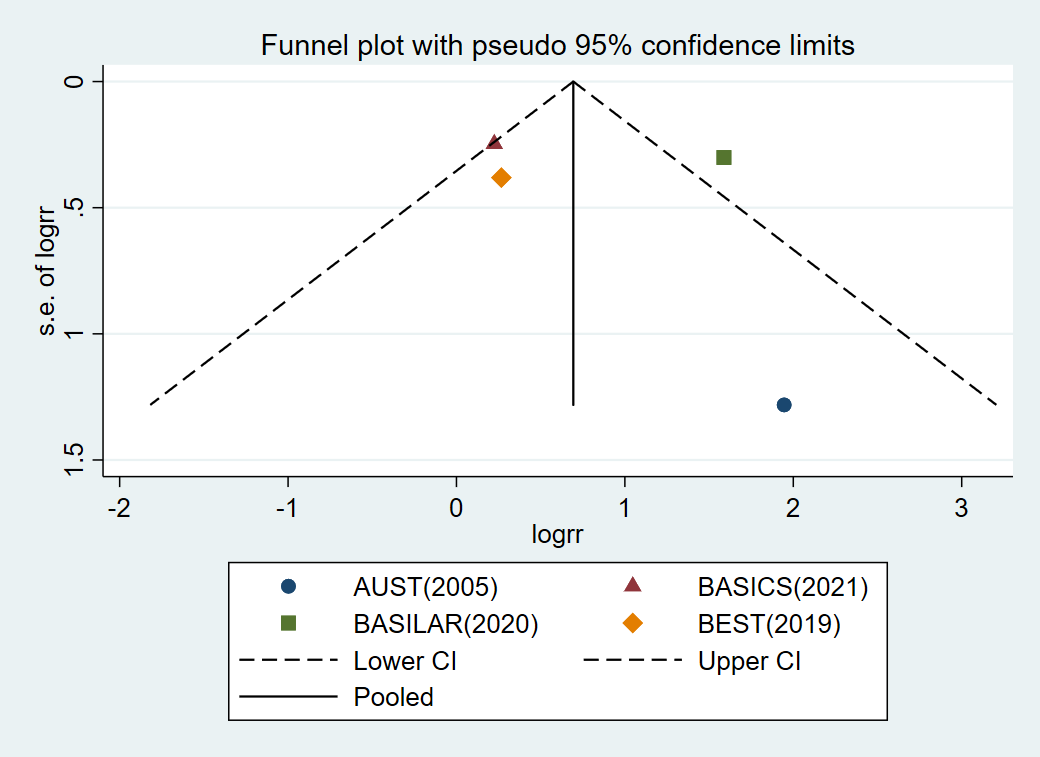

Supplement: Supplementary file 1 [file Table_1.docx]
